# Specific olfactory neurons and glomeruli are associated to differences in behavioral responses to pheromone components between two *Helicoverpa* species

**DOI:** 10.3389/fnbeh.2015.00206

**Published:** 2015-08-04

**Authors:** Han Wu, Meng Xu, Chao Hou, Ling-Qiao Huang, Jun-Feng Dong, Chen-Zhu Wang

**Affiliations:** ^1^State Key Laboratory of Integrated Management of Pest Insects and Rodents, Institute of Zoology, Chinese Academy of SciencesBeijing, China; ^2^Department of Plant Protection, Forestry College, Henan University of Science and TechnologyLuoyang, China

**Keywords:** behavior, sex pheromone, olfactory sensory neurons, calcium imaging, antennal lobe

## Abstract

Sex pheromone communication of moths helps to understand the mechanisms underlying reproductive isolation and speciation. *Helicoverpa armigera* and *Helicoverpa assulta* use (Z)-11-hexadecenal (Z11-16:Ald) and (Z)-9-hexadecenal (Z9-16:Ald) as pheromone components in reversed ratios, 97:3 and 5:95, respectively. *H. armigera* also produces trace amount of (Z)-9-tetradecenal (Z9-14:Ald) in the sex pheromone gland, but *H. assulta* does not. Wind tunnel studies revealed that the addition of small amounts (0.3%) of Z9-14:Ald to the main pheromone blend of *H. armigera* increased the males' attraction, but at higher doses (1%, 10%) the same compound acted as an inhibitor. In *H. assulta*, Z9-14:Ald reduced male attraction when presented as 1% to the pheromone blend, but was ineffective at lower concentrations (0.3%). Three types (A–C) of sensilla trichodea in antennae were identified by single sensillum recording, responding to Z11-16:Ald, Z9-14:Ald, and both Z9-16:Ald and Z9-14:Ald, respectively. Calcium imaging in the antennal lobes (ALs) revealed that the input information of the three chemicals was transmitted to three units of the macroglomerular complex (MGC) in ALs in both species: a large glomerulus for the major pheromone components, a small one for the minor pheromone components, and a third one for the behavioral antagonists. The type A and C neurons tuned to Z11-16:Ald and Z9-16:Ald had a reversed target in the MGC between the two species. In *H. armigera*, low doses (1, 10 μg) of Z9-14:Ald dominantly activated the glomerulus which processes the minor pheromone component, while a higher dose (100 μg) also evoked an equal activity in the antagonistic glomerulus. In *H. assulta*, instead, Z9-14:Ald always strongly activated the antagonistic glomerulus. These results suggest that Z9-14:Ald plays different roles in the sexual communication of two *Helicoverpa* species through activation of functionally different olfactory pathways.

## Introduction

To mate successfully, male moths have to correctly identify sex pheromones released by conspecific females. In most moth species, the pheromones contain two or a few chemical components in a species-specific ratio (Arn et al., 1992; Mustaparta, 1997; Berg et al., 2014). Behavioral studies have demonstrated that both individual conspecific and heterospecific sex pheromone components may act as behavioral antagonists when they are released at inappropriate ratios and rates (Vetter and Baker, 1984; Vickers et al., 1991; Baker, 2008). However, the olfactory mechanisms of the antagonist effect remains poorly understood.

Sex pheromone components are first detected by olfactory sensory neurons (OSNs) in the sensilla trichodea on antennae of male moths (Hansson et al., 1987; Kaissling et al., 1989; Cossé et al., 1998; Baker et al., 2004). Then the pheromone signals are transmitted to the first olfactory processing center, the antennal lobe (AL). In the AL, the OSNs expressing the same odor receptor converge to the same glomerulus (Gao et al., 2000; Vosshall et al., 2000). Male Heliothine moths possess a special and large macroglomerular complex (MGC), which usually consists of three or four glomeruli and is dedicated to processing the intra- and interspecific pheromone information, as demonstrated by electrophysiological recording combined with staining of AL neurons (Hansson et al., 1991; Vickers et al., 1998; Berg et al., 2005). For several Heliothine species, two essential sex pheromone components of respective pheromone blends are represented in two separate MGC glomeruli, and at least one other glomerulus of the MGC is used to process known behavioral antagonists (Hansson et al., 1995; Vickers et al., 1998; Vickers and Christensen, 2003; Lee et al., 2006a,b; Zhao and Berg, 2010).

The two sympatric and closely-related species, *Helicoverpa armigera* and *Helicoverpa assulta* both use (Z)-11-hexadecenal (Z11-16:Ald) and (Z)-9-hexadecenal (Z9-16:Ald) as essential sex pheromone components but in opposite ratios (Kehat et al., 1980; Cork et al., 1992). Behavioral studies have demonstrated that the binary blends of Z11-16:Ald and Z9-16:Ald at their pheromone gland ratios (97:3 in *H. armigera*, 7:93 in *H. assulta*) are sufficient for eliciting male attraction behavior in the corresponding species (Zhao et al., 2006; Ming et al., 2007). Besides Z11-16:Ald and Z9-16:Ald, about 0.25–1.5% (Z)-9-tetradecenal (Z9-14:Ald) was found in the female pheromone gland of *H. armigera* but not in that of *H. assulta* (Kehat and Dunkelblum, 1990; Zhang et al., 2012). There are conflicting results on the effect of addition of Z9-14:Ald to the pheromone blends in previous studies. Field tests in Australia demonstrated that captures of *H. armigera* males were significantly increased with the addition of 2–5% Z9-14:Ald to Z11-16:Ald (Rothschild, 1978). In China, behavioral data showed that 0.3% Z9-14:Ald in combination with binary pheromone blends trapped more *H. armigera* males in field tests (Zhang et al., 2012). However, wind tunnel experiments and field traps in Israel showed that 5–10% Z9-14:Ald in the synthetic pheromone blend significantly inhibited the attraction behavior of *H. armigera* males (Gothilf et al., 1978; Kehat and Dunkelblum, 1990). In the Korean population of *H. assulta* the addition of Z9-14:Ald interrupted the pheromone-mediated communication resulting in a significant reduction in trap catches (Boo et al., 1995).

In this study, we first investigated the behavioral responses of the two *Helicoverpa* species to a series of synthetic blends that contained different amounts of Z9-14:Ald added to the binary pheromone blends of the corresponding species. In order to explain how Z9-14:Ald plays different roles in the pheromone communication of two *Helicoverpa* species, we further characterized the related OSNs in antennae and response patterns in antennal lobes, and revealed different olfactory pathways activated by Z9-14:Ald in males of the two species.

## Materials and methods

### Insects

For *H. armigera* and *H. assulta* are agricultural pests in China, no specific permission was required to collect any of these samples. All insect samples were treated according to the guideline of State Key Laboratory of Integrated Management of Pest Insects and Rodents, Institute of Zoology, Chinese Academy of Sciences. *H. armigera* and *H. assulta* larvae were collected from tobacco field in Zhengzhou, Henan Province, China, and reared in the laboratory on an artificial diet (Wu and Gong, 1997). Each year, some individuals of the second generation from the field are introduced into the laboratory colonies. The successive generations were maintained in an environmental chamber at 26 ± 1°C with a relative humidity of 40%–60% on a 16L:8D photoperiod. Pupae of different sex were separated daily. Adult male moths used in the physiological and behavioral experiments were 2–5 days old.

### Chemicals

Z11-16:Ald, Z9-16:Ald, and Z9-14:Ald were purchased from Shin-Estu company (Tokyo, Japan). The compounds were further purified to >98% by column chromatography on silica gel. Series of dilutions of the pheromones in redistilled paraffin oil (Analytical grade, Fluka) were stored in 2 ml glass vials (Agilent, Technologies, USA) at -20°C.

### Behavioral tests

A wind tunnel was used to investigate the role of Z9-14:Ald in the attraction behavior of *H. armigera* and *H. assulta* males. In *H. armigera*, the following odor sources were tested: (1) hexane, used as the negative control for it was the solvent of pheromone components; (2) binary mixture of Z11-16:Ald and Z9-16:Ald at the pheromone gland ratio of 97:3, used as the positive control for *H. armigera* (PC1); (3) PC1+0.3% Z9-14:Ald; (4) PC1+0.5% Z9-14:Ald; (5) PC1+1% Z9-14:Ald; (6) PC1+10% Z9-14:Ald. In *H. assulta*, hexane was also used as the negative control and the binary mixture of Z11-16:Ald and Z9-16:Ald at the pheromone gland ratio of 5:95 as the positive control (PC2). We also tested the males responses to both PC2+0.3% Z9-14:Ald and PC2+1% Z9-14:Ald. Each odor source was loaded on a rubber septum. The loaded dosage for the positive control was 1 mg (10 μl of 100 μg/μl solution). The proportion of Z9-14:Ald was relative to the amount of PC1 or PC2. For example, PC1+ 0.3% Z9-14:Ald in *H. armigera* means 1 mg of the binary mixture of Z11-16:Ald and Z9-16:Ald at 97:3 in combination with 3 μg Z9-14:Ald. Different amounts of Z9-14:Ald were added to reach the established proportions. In each experiment, 9–15 male moths were tested on any given day. The order of treatments in each replicate was randomized. At least three replicates were run.

Behavioral observations for males of the two *Helicoverpa* species were performed in a wind tunnel as described previously (Zhao et al., 2006). Briefly, 2–5 days old virgin males were exposed during their scotophase (4–6 h) in a clear plexiglass wind tunnel (2.5 m long, 1 m wide, 1 m high). The males were transferred to the wind tunnel room ca. 45 min prior to observations in order for them to become acclimated to the room conditions (0.3–0.5 m/s wind velocity, 24 ± 2°C, 40–60% relative humidity, 0.3 Lux red light). The males responses were categorized according to the following stereotyped behavior (1) Flight: taking off from release cage; (2) Upwind: flying at the height of lure and showing characteristic zig–zag paths toward pheromone source; (3) Close: continuing upwind behavior and flying within 10 cm of pheromone source; (4) Landing: contacting the lure.

### Physiological characterization of the OSNs responding to Z11-16:Ald, Z9-16:Ald and Z9-14:Ald, and their corresponding glomeruli

We used the cut-sensillum technique *in vivo* to physiologically characterize the selectivity of OSNs in male antennae of *H. armigera* and *H. assulta* to Z11-16:Ald, Z9-16:Ald, and Z9-14:Ald. Paraffin oil was used as the solvent of these chemicals because it induced no response in both SSR and calcium imaging experiments. The responding OSNs were located on the flagellar segments number 25–60 in the antenna. To determine the selectivity of OSNs, we used the dosage of 100 μg of each compound (10 μl of 10 μg/μl solutions) as test stimulus. The dosage chosen was based on the dose response curves of OSNs in our previous work (Wu et al., 2013), in which about 10 μg Z11–16:Ald or Z9–16:Ald dissolved in hexane could induce 50% of each OSN maximal firing rate. We did a preliminary experiment which indicated that 10 μg Z11-16:Ald or Z9-16:Ald dissolved in hexane (10 μl) induced an equal response with 100 μg of corresponding compounds in paraffin oil (10 μl). Paraffin oil was used as a control. We also tested the dose responses of Z9-14:Ald with different doses (0.1, 1, 10, 100, 1000 μg, each diluted in 10 μl paraffin oil) in both species. The cross-adaptation experiments between Z9-16:Ald and Z9-14:Ald were carried out to determine whether the different spiking activity could be attributed to the same neuron or to the different neurons in the same sensillum (Cossé et al., 1998; Baker et al., 2004; Domingue et al., 2010).

*In vivo* calcium imaging was used to reveal the functional relationship between the OSNs tuned to Z11-16:Ald, Z9-16:Ald, and Z9-14:Ald and the targeted MGC subunits in the ALs of *H. armigera* and *H. assulta* males. Paraffin oil was used as control. The order of the stimuli was randomized. Because males showed to be sensitive to the addition of trace amount of Z9-14:Ald in behavior experiments, this compound was used in three doses (1, 10, 100 μg each diluted in 10 μl paraffin oil) for optical recordings. The methods how to recognize the activated glomeruli were described in detail in our previous work (Wu et al., 2013). Briefly, we first identified the activated area from false-color coded images in the calcium imaging. Then we superimposed the response pattern of the odorant (>50% activity) on gray scale images of the AL by ImageJ software with Z project treatment. Because the AL atlas of the two *Helicoverpa* species are available (Berg et al., 2002; Skiri et al., 2005), we identified the activated glomerulus of the MGC according to their position and outline.

### Single sensillum recording

The cut-sensillum technique was conducted as described earlier (Kaissling, 1974; Baker et al., 2004). Briefly, the male moth was first placed inside a 1-ml pipette tip with the narrow end cut to ensure the head to pass through. Then the head with protruding antennae was fixed on a fastening device. A tungsten electrode was inserted into one of the compound eyes to serve as a ground connection. The sensilla were randomly selected mainly from the 25th to the 60th flagellar segment. After cutting the sensillum tip with home-made forceps, the spiking activities of OSNs were collected through an Ag-AgCl recording electrode connected to an amplifier. For each compound, 10 μl of 10 μg/μl solution were placed on a filter paper strip (0.7 cm × 2.5 cm) held in a Pasteur pipette (15 cm long).

A continuous stream of purified and humidified air was directed on the antenna (12.5 ml/s) from the outlet of a steel tube (i.d. 6 mm, length 15 cm) positioned 2 cm away. Test odors were injected into the air stream using a stimulus flow-controller (CS-55, Syntech), which generated air pulses through the odor cartridge at a flow rate of 10 ml/s. The stimulus time was 200 ms and the sensilla were grouped according to the response profiles of OSNs. The signals from the recording electrode were sent to a programmable signal-recording and output controller (IDAC-4, Syntech, Hilversum, Netherlands). Autospike version 3.4 software (Syntech) was used for both recording and data analysis. In the same sensillum, action potentials from OSNs were declared to be “larger” or “smaller” on the base of their spike size. Action potential frequencies (spikes/s) were calculated by counting the number of spikes occurring during the first 200 ms of the spike train initiated by a stimulus puff. The cross-adaptation experiments between Z9-16:Ald and Z9-14:Ald were conducted using the method described previously (Cossé et al., 1998; Baker et al., 2004; Domingue et al., 2010).

### *In vivo* calcium imaging

Animal preparations were performed as reported in the literature (Hansson et al., 2003; Galizia and Vetter, 2005). After dissection, a calcium-sensitive dye CaGR-1-AM (Molecular Probes, Eugene, OR, USA) was used to stain the brain. Then the insect was placed into a dark room at 12°C. One hour later, the brain was thoroughly rinsed with Ringer solution (Christensen and Hildebrand, 1987) and ready for imaging. The stimuli were applied with the same method used for single sensillum recording.

Imaging data were collected with a Till-Photonics imaging system (Till Photonics, Germany) in combination with an epifluorescent microscope (Olympus BX-51WI, Tokyo, Japan) equipped with a 20 × (NA 0.95, Olympus) water immersion objective. A stimuli sequence contained 40 frames with a sampling rate of 4 Hz. Exposure time was 200 ms for each frame and stimulation was set at frame 12 and lasted for 500 ms. To increase signal to noise ratio, images were binned 2 × on chip (to 320 × 240 pixels).

Raw data were filtered using spatial and time median filters to remove “salt and pepper” noise (Galizia and Vetter, 2005). Background fluorescence (F) was defined as the mean fluorescence of frames 2–11, recorded just before onset of stimulation. For the false color images, the fluorescence change of each frame was expressed as ΔF/F. In time traces, Fn/F (*n* = 1–40) was first expressed for the ratio of each frame against background and then followed by a smooth treatment (smooth, 30 frames). ΔF/F of each frame was defined as the difference between the signals before and after smoothing, i.e., ΔF/F = Fn/F–Fn/F (smooth, 30 frames). Next, each pixel was assigned a value that was then converted into a color. Smooth treatment can reduce noise but not remove pertinent signals. For quantitative analysis of the response intensity, activity foci with averaged area of 177 pixels were chosen to represent the glomerular response after recognition of targeted glomerulus. The mean of three sequential frames at the signal's maximum was taken as the amplitude of odor-induced responses.

### Data analyses

In wind tunnel experiment, percents of males performing sequential behaviors were subjected to Chi-square test of independence with Yates' continuity correction (*P* < 0.05). Data of single sensillum recording were analyzed by the One-Way ANOVA for analysis of variance, and the least significant difference (LSD) test was used for means multiple comparisons (*P* < 0.05). In calcium imaging, data were acquired by the software Till-vision (Till photonics) and further analyzed by software ImageJ (NIH, USA) and custom-made programs in MATLAB (The Math Works, Inc). Paired *t*-tests were used to compare the sensitivity between subunits of MGC (*P* < 0.01).

## Results

### Behavioral response of males to different synthetic blends

**Figure 1:**
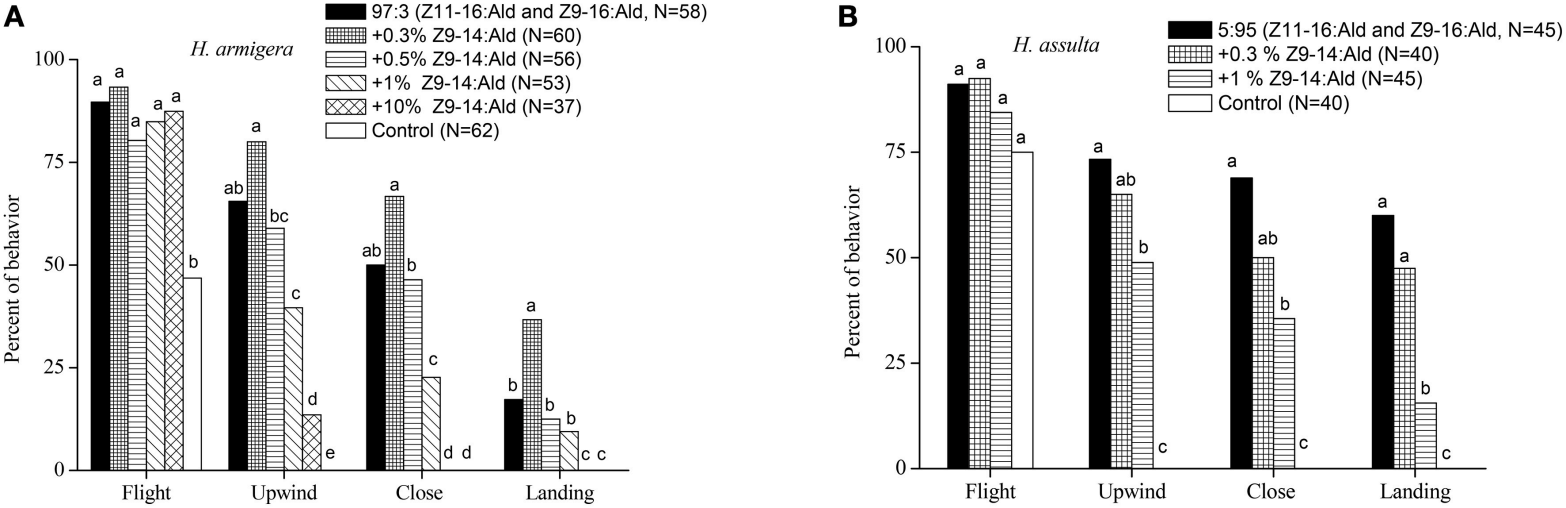
**Behavioral responses males of ***Helicoverpa armigera*** (A) and ***H. assulta*** (B) to a series of synthetic blends**. The blends contained varying dosages of Z9-14:Ald relative to the binary mixture (Z11-16:Ald and Z9-16:Ald at gland ratios of each species). Hexane was used as control. Symbols in the same behavioral category bearing different letters are significantly different (Chi-square test, *P* < 0.05).

In the wind tunnel, males of *H. armigera* were highly attracted to the binary pheromone blend (Z11-16:Ald and Z9-16:Ald) at the gland ratio (97:3) and showed robust mate-seeking behavior (Figure 1A). The presence of Z9-14:Ald in the ternary blend at a relatively low ratio (0.3%) significantly increased the males landing behavior. Increasing Z9-14:Ald to 0.5% had no significant effect on males' behavioral responses. Instead, higher doses (1% and 10%) of Z9-14:Ald in the ternary blends did not affect taking flight, but inhibited the males upwind and close searching behavior. Therefore, Z9-14:Ald could act as agonist or antagonist to *H. armigera* males, depending on its concentration (Figure 1A).

Males of *H. assulta* also exhibited stereotypical attraction behavior to the binary pheromone blend (Z11-16:Ald and Z9-16:Ald) at gland ratio (Figure 1B), and the addition of 0.3% Z9-14:Ald in the pheromone blend did not significantly affect male attraction. However, in the presence of 1% Z9-14:Ald, *H. assulta* males were less attracted to the ternary blend (Figure 1B).

### Olfactory sensory neurons (OSNs) tuned to Z11-16:Ald, Z9-16:Ald, and Z9-14:Ald

**Figure 2:**
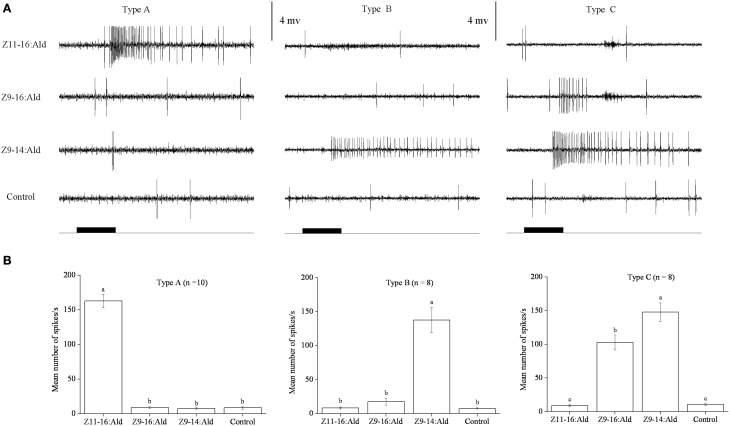
**Three types of trichoid sensilla in the antennae of male ***Helicoverpa armigera*****. **(A)** Typical response profiles of olfactory sensory neurons (OSNs) housed in three types of sensilla. Type A and type B sensilla contain one OSN tuned to Z11–16:Ald and Z9-14:Ald, respectively; type C sensilla contain OSNs responding to both Z9-16:Ald and Z9-14:Ald. **(B)** Mean firing rate of OSNs within two types of sensilla trichodea. Firing rate (spikes/s) was calculated by counting the actual number of spikes occurring during the first 200 ms of the response. All compounds were used at the dose of 100 μg. Paraffin oil was used as control. Values are Mean ± SEM. Bars marked with the same letters are not significantly different from one another (One-Way ANOVA followed by LSD test, *P* < 0.05).

**Figure 3:**
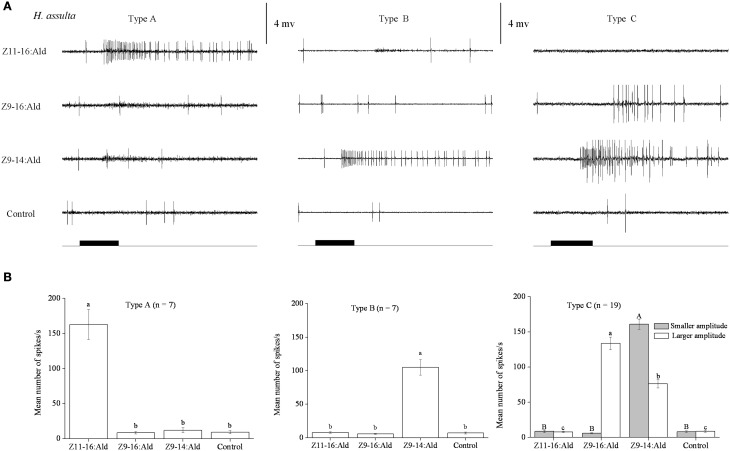
**Three types of trichoid sensilla in the antennae of male ***Helicoverpa assulta***. (A)** Typical response profiles of olfactory sensory neurons (OSNs) housed in three types of sensilla. Type A and type B sensilla contain one kind of OSN tuned to Z11–16:Ald and Z9–14:Ald, respectively. In type C sensilla of *H. assulta*, there are two kinds of OSNs, the one with larger amplitude responds to both Z9-16:Ald and Z9-14:Ald, the other with smaller amplitude responded only to Z9-14:Ald. **(B)** Mean firing rate of OSNs within three types of sensilla trichodea. Firing rate (spikes/s) was calculated by counting the actual number of spikes occurring during the first 200 ms of the response. All compounds were used at the dose of 100 μg. Paraffin oil was used as control. Values are Mean ± SEM. Bars marked with the same letters are not significantly different from one another (One-Way ANOVA followed by LSD test, *P* < 0.05).

In our SSR experiments, we detected three distinctly different response profiles in both *H. armigera* and *H. assulta* (Figures 2, 3). Here, the three types of sensilla are named according to what has been reported for other Heliothine species (Baker et al., 2004). The type A sensilla contained OSNs responding only to Z11-16:Ald, those of type B were specifically tuned to Z9-14:Ald, while the OSNs of type C sensilla could be activated by both Z9-16:Ald and Z9-14:Ald (Figures 2, 3).

**Figure 4:**
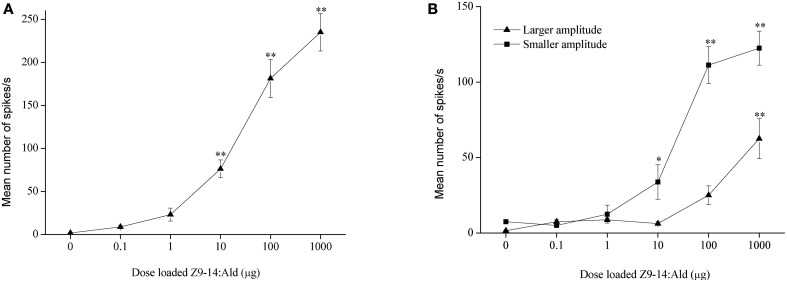
**Dose response profiles of olfactory sensory neurons tuned to Z9-14:Ald in the type C sensilla. (A)** *H. armigera, n* = 8, **(B)** *H. assulta, n* = 4. Paraffin oil was used as control. Values are Mean ± SEM (One-Way ANOVA, ^*^P < 0.05; ^**^P < 0.01).

In *H. armigera*, out of 451 responding sensilla, 399 were of type A (88%), 9 were of type B (2%), and 43 were of type C (10%) (Figure 2). In the type C Z9-14:Ald and Z9-16:Ald evoked distinct responses at the dosage of 100 μg (Figure 2B). Based on spike amplitude, it appears that the same OSN in the type C sensilla responds to both compounds (Figure 2A). The dose response curve of Z9-14:Ald in the type C sensilla showed an increase in firing rate in a dose-related manner and from 10 μg Z9-14:Ald induced significant spiking activities (Figure 4A).

However, we found two different response profiles in type C sensilla when stimulated with Z9-16:Ald and Z-11-hexadecenol (Z11-16:OH), suggesting there are at least two different OSNs in this type of sensilla (Figure S1 in Supplementary Material). In the following cross-adaptation experiments, we found that regardless of whether Z9-16:Ald or Z9-14:Ald was the first stimulus, the OSN was partially adapted by the second stimulation with the same odorant (Figure S2 in Supplementary Material). A similar pattern was found when a first stimulation with Z9-14:Ald was followed by one with Z9-16:Ald (Figure S2 in Supplementary Material). However, one OSN was not adapted when we first stimulated with Z9-16:Ald and then with Z9-14:Ald, indicating the presence of two different OSNs responding to Z9-14:Ald in the type C sensillum of *H. armigera* (Figure S2 in Supplementary Material).

In *H. assulta*, out of 182 responding sensilla, 11 and 10 were classified as type A (6%) and B (5%), respectively, while 161 belonged to type C (89%) (Figure 3). Based on spike amplitude, we could clearly distinguish two OSNs in each sensillum of type C (Figure 3A). The first with the larger spike amplitude responded to both Z9-16:Ald and Z9-14:Ald, with a stronger signal for the first compound when the stimuli were used at a dose of 100 μg (Figure 3B). The second OSN responded only to Z9-14:Ald (Figure 3). The dose-response curves showed that the dosage for Z9-14:Ald, which elicited response in the large-spiking OSNs was 100 times higher than that for the smaller-spiking one (Figure 4B).

### MGC glomeruli activated by Z9-14:Ald in two *Helicoverpa* species

**Figure 5:**
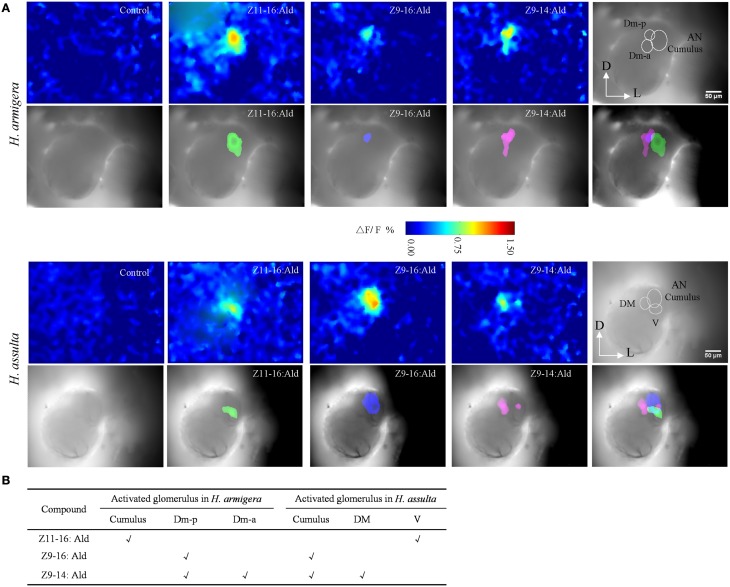
**Spatial representation of Z11-16:Ald, Z9-16:Ald, and Z9-14:Ald in the antennal lobe (AL)**. **(A)** Activated patterns of Z11-16:Ald, Z9-16:Ald, and Z9-14:Ald in the ALs of *H. armigera* and *H. assulta*. For each species, the upper panels show false-color-coded spatial response patterns of the three chemicals, with the glomerular structure in the last picture (D, dorsal; L, lateral). The lower panels show the activated patterns (exceeding 50% of the maximum activity) of the three chemicals are superimposed on the gray-scale images with the last image indicating the relative positions of the activated glomeruli. **(B)** The glomerulus activated by Z11-16:Ald, Z9-16:Ald, and Z9-14:Ald, according to the AL atlas for the tested species.

Based on the AL atlas of *H. armigera* and *H. assulta* (Berg et al., 2002; Skiri et al., 2005), we were able to identify the activated MGC subunits according to their position and outline. The activity patterns (frontal view) shown in Figure 5 were evoked by stimulation with 100 μg of Z11-16:Ald, Z9-16:Ald, and Z9-14:Ald.

In *H. armigera*, the largest glomerulus (cumulus) was activated by Z11-16:Ald and the posterior dorsomedial unit (Dm-p) by Z9-16:Ald (Figure 5A), which is consistent with our previous results (Wu et al., 2013). Both the Dm-p and the anterior dorsomedial unit (Dm-a) were simultaneously activated by Z9-14:Ald (Figure 5A) and the spatial representation in the AL was reproducible among different individuals.

In *H. assulta*, the cumulus was activated by Z9-16:Ald and the ventral unit (V) by Z11-16:Ald. Z9-14:Ald evoked a strong response in the dorsomedial unit (DM) and a weak response in the cumulus (Figure 5A). In some preparations, Z9-14:Ald did not induce any response in the cumulus.

**Figure 6:**
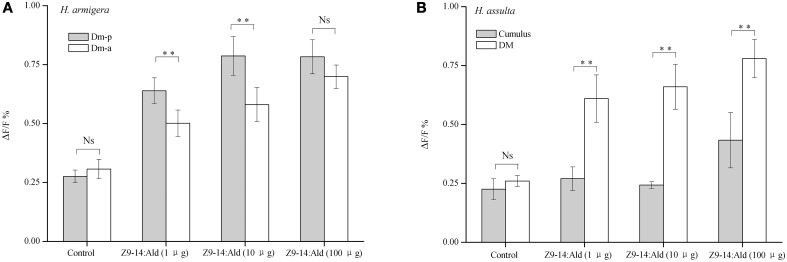
**Glomerular activities induced by Z9-14:Ald at different dosages in the two ***Helicoverpa*** species**. The tested dosages were 1, 10, 100 μg. **(A)** Comparison of glomerular activities between the Dm-p and the Dm-a in *H. armigera* (*n* = 9). **(B)** Comparison of glomerular activities between the DM and the cumulus in *H. assulta* (*n* = 6). Values are Mean ± SEM (Paired *t*-test, ^**^P < 0.01, ^Ns^ No significant difference).

Higher concentrations of Z9-14:Ald induced correspondingly stronger responses in ALs of both *Helicoverpa* species (Figure 6). In *H. armigera*, Z9-14:Ald activated both the Dm-p and the Dm-a in the MGC of the AL (Figures 5, 6). The Dm-p was also activated by Z9-16:Ald, which is the secondary sex pheromone component of *H. armigera*. The Dm-p was significantly more sensitive than the Dm-a to Z9-14:Ald at the dosages of 1μg and 10 μg. However, the Dm-p and the Dm-a showed similar activities to Z9-14:Ald at the dosage of 100 μg (Figure 6A).

In *H. assulta*, 1 or 10 μg of Z9-14:Ald only evoked responses in the DM, but when used at the dose of 100 μg this compound, besides inducing a strong response in the DM also produced a weak signal in the cumulus (Figures 5A, 6B). Statistical analysis of the results showed that the DM was much more sensitive than the cumulus to Z9-14:Ald all across the range of tested doses (Figure 6B).

## Discussion

Z11-16:Ald and Z9-16:Ald are the essential components of pheromone blends for both *H. armigera* and *H. assulta* but in reverse ratios, 97:3 and 5:95, respectively. In the present study, Z9-14:Ald acted as an agonist when minor amounts were added to the binary pheromone blend of *H. armigera*, but as an antagonist at a higher levels. For *H. assulta*, Z9-14:Ald acted as an antagonist especially when presented with higher concentration. Based on the activated OSNs and related glomeruli, we found that the agonist and the antagonist effect of Z9-14:Ald involved different olfactory pathways in the two species.

### OSNs and related glomeruli mediating attraction and aversion

Sex pheromones are first detected by OSNs on the antennae and then the signals are sent to the MGC of the AL via a labeled-line system, as exemplified in many moth species (Christensen and Hildebrand, 1987; Vickers and Christensen, 2003; Berg et al., 2005; Lee et al., 2006a,b). Comparative and phylogenetic studies have demonstrated that several Heliothine moth species are closely-related and share a common organization and information processing in the olfactory system. The MGC is generally composed of 3-4 glomeruli, two of them are used to process sex pheromones and at least one glomerulus is dedicated to processing known behavioral antagonists (Vickers et al., 1998; Berg et al., 2005; Baker, 2008).

**Figure 7:**
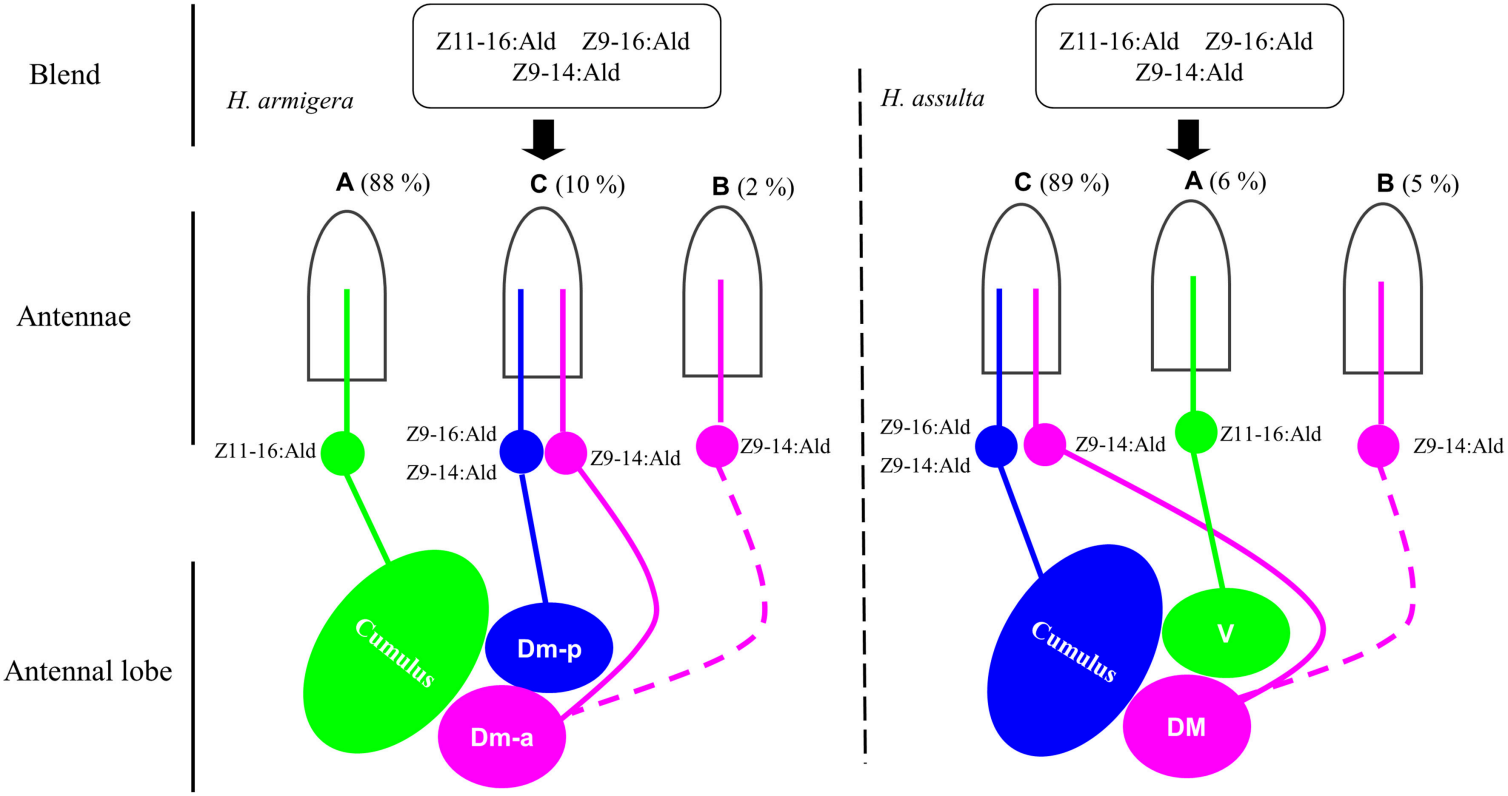
**Drawing based on different combinations of special olfactory pathways encoding different behaviors**. The picture drawn based on the results from single sensillum recording, calcium imaging and behavioral tests. Green: the neurons tuned to Z11-16:Ald and the target glomerulus. Blue: the neurons tuned to Z9-16:Ald and Z9-14:Ald and the target glomerulus. Pink: the neurons tuned to Z9-14:Ald and the target glomerulus. Hatched line: the speculated pathway. Dm-p, posterior dorsomedial unit; Dm-a, anterior dorsomedial unit; DM, dorsomedial unit; V, ventral unit.

In *H. armigera*, both Z9-16:Ald and Z9-14:Ald activated Dm-p, while Z9-14:Ald also activated Dm-a (Figure 5). Based on our SSR results and the hypothesis that the OSNs expressing the same odorant receptor converge onto the same glomerulus, we predict that there are two compartmentalized OSNs in type C sensillum of *H. armigera*, in which one is tuned to both Z9-16:Ald and Z9-14:Ald and another only to Z9-14:Ald (Figures S1, S2 in Supplementary Material and Figure 7). This topography of type C in *H. armigera* is similar to that of the closely-related species *Helicoverpa zea* (Cossé et al., 1998; Lee et al., 2006a). In *H. assulta*, Z9-14:Ald induced a strong response in the DM and a weak response in the cumulus (Figures 5, 6), in agreement with the observation that the large-spike OSN of type C is tuned to both Z9-16:Ald and Z9-14:Ald, while the small-spike OSN responds only to Z9-14:Ald (Figures 3, 7).

Each of the two principal sex pheromone components activated single glomeruli of the MGC in *H. armigera* and *H. assulta* (Wu et al., 2013). In *H. armigera*, Z11-16:Ald and Z9-16:Ald activate the cumulus and the Dm-p of the MGC, respectively (Figure 5), while in *H. assulta*, Z11-16:Ald and Z9-16:Ald evoked a response in the ventral unit and in the cumulus of the MGC, respectively (Figure 5). Based on the structure and functional significance of MGC glomeruli in these two species (Berg et al., 2002; Skiri et al., 2005), we confirm that the Dm-a in *H. armigera* and the DM in *H. assulta* are involved in processing behavioral antagonists (Figure 7). *H. zea*, a most closely-related species with *H. armigera*, has a similar MGC structure in which the Dm-a is used to process behavioral antagonists (Lee et al., 2006a).

Z9-14:Ald activates the glomeruli processing behavioral antagonists, Dm-a in *H. armigera* and DM in *H. assulta*, but why does Z9-14:Ald act as agonist in *H. armigera* and increases males' attraction at lower dosages? Dose responses of Z9-14:Ald in the AL of *H. armigera* show that the Dm-p is more sensitive than the Dm-a when presented with low dosages of Z9-14:Ald (1 and 10 μg, Figure 6A), but both respond with similar intensity to higher doses of the same stimulus (100 μg, Figure 6A). Therefore in *H. armigera*, low dosages of Z9-14:Ald in the pheromone blends act as an agonist and activate the more sensitive glomerulus, the Dm-p, which is responsible for the principal pheromone component Z9-16:Ald, but does not reach the threshold for activating the Dm-a responsible for behavioral antagonists. Instead, higher dosages of Z9-14:Ald activate both the Dm-p and the Dm-a, leading to the interruption of attraction behavior (Figure 7). For *H. assulta*, the antagonistic DM was significantly more sensitive than the cumulus to Z9-14:Ald at any tested dosage (Figure 6B). Such a difference in sensitivity of the two glomeruli could be due to the difference in sensitivity of two OSNs responding to Z9-14:Ald in the type C sensilla (Figure 4B). Previous behavioral and neurophysiological data demonstrated that the DM is involved in processing behavioral antagonists (Boo et al., 1995; Berg et al., 2005; Zhao and Berg, 2010). This can explain why the addition of Z9-14:Ald to the pheromonal blend results in the antagonism of upwind flight in *H. assulta* males (Figure 7). In *Drosophila* species, it has been demonstrated that the same compounds at different concentrations activate different neurons and related glomeruli and finally elicit different behaviors, thus confirming that different stimulus intensities can evoke distinct perception (Semmelhack and Wang, 2009; Lin et al., 2013; Zhang et al., 2013).

### The abundance of OSNs tuned to Z9-14:Ald on the antennae

In general, when the OSNs tuned to sex pheromone components are in different sensilla, the abundance of the related sensilla corresponds to the ratio of the components in the pheromonal blend (Cossé et al., 1998; Baker et al., 2004; Wu et al., 2013). However, although Z9-14:Ald is not a sex pheromone component in *H. assulta*, and not the principal one in *H. armigera*, we found that the sensilla responding to this compound account for about 94% in *H. assulta* (including 5% type B sensilla and 89% type C sensilla) and 12% (2% type B and 10% type C sensilla) in *H. armigera*. This characteristic seems to be common among heliothine moths. In fact, there are about 29 and 26% sensilla housing OSNs tuned to the non-pheromone component Z9-14:Ald in *H. zea* and *Heliothis subflexa*, respectively (Cossé et al., 1998; Baker et al., 2004). Z9-14:Ald is an essential pheromone component for North-American *Heliothis virescens* (Klun et al., 1980), but it is an antagonist and decreases male attraction for the sympatric moth *H. zea* (Shaver et al., 1982; Vickers et al., 1991). Z9-14:Ald is also a sex pheromone component of *Heliothis peltigera* (Dunkelblum and Kehat, 1989). These phenomenon suggests that Z9-14:Ald plays an important role in pheromone communication and also in behavioral isolation of the sympatric heliothine species.

Z9-14:Ald may be an evolutionarily old sex pheromone component of ancestral heliothine species, and thus the OSNs tuned to Z9-14:Ald would be also primitive in this group of insects. During speciation such OSNs could adapt to other related chemicals with maintenance of their original trait. Molecular phylogenetics suggest that *H. virescens* and *H. peltigera* using Z9-14:Ald as sex pheromone components are ancestral compared to *H. armigera* and *H. assulta* (Cho et al., 1995, 2008), in which the OSNs of type C sensilla keep a higher sensitivity to Z9-14:Ald, meanwhile respond to their pheromone component, Z9-16:Ald (Figures 2, 3).

## Concluding remarks

Our study clarifies that Z9-14:Ald serves as both agonist and antagonist in sex pheromone communication of *H. armigera* depending on its relative ratio, but acts as an antagonist in *H. assulta*. Such behavioral shifts in the two species result from that Z9-14:Ald activates different specific combinations of OSNs and related glomeruli in the MGC. Functional divergence in pheromone-related olfactory pathways helps to shape pheromone perception, contributing to understand the mechanisms of reproductive isolation and speciation. In the antennal lobe of moth species, the projection neurons receive input from OSNs and send output to the higher brain centers, such as the mushroom body and the lateral horn. Future research will focus on the mechanism by which these centers generate different behaviors.

## Author contributions

HW: Designed the experiments, provided and analyzed experimental data, and wrote the manuscript. MX: Provided experimental data. CH: Provided experimental data. LQH and JFD: Provided materials and technical support on experiments. CZW: Conceived the project, advised with experimental procedures, analyzed data, and wrote the manuscript.

### Conflict of interest statement

The authors declare that the research was conducted in the absence of any commercial or financial relationships that could be construed as a potential conflict of interest.

## Supplementary material

Figure S1 **Two types of neurons colocalized within type C sensilla in ***Helicoverpa armigera***. (A)** Response profile of type C sensilla to Z9-16:Ald (100 μg), Z11-16:OH (100 μg), and Z9-14:Ald (1, 10, 100 μg). Paraffin oil was used as control. The horizontal black bar indicates stimulus duration of 200 ms. (**B**) The wave forms of spikes sampled during 10 s of spontaneous activity (same neuron as in A responding to control) indicates two neuron types. (**C**) Amplitude classification of firing patterns during 10 s showing that Z9-16:Ald activated the larger-spike neuron, Z11-16:OH activated the smaller-spike neuron, while Z9-14:Ald at the dosage 100 μg activated both neurons.

Figure S2 **Examples of traces generated during cross-adaptation stimulation of an OSN in a ***H. armigera*** Type C sensillum**. *Horizontal* bar denotes stimulus delivery time of 100 ms. The interval between two stimulations is about 350 ms. All stimuli loadings were of 100 μg.
