# Supplementary figures and images for: Specific olfactory neurons and glomeruli are associated to differences in behavioral responses to pheromone components between two Helicoverpa species
Source: Front Behav Neurosci. 2015 Aug 4;9:206. doi: 10.3389/fnbeh.2015.00206 (PMC4523827; doi:10.3389/fnbeh.2015.00206)

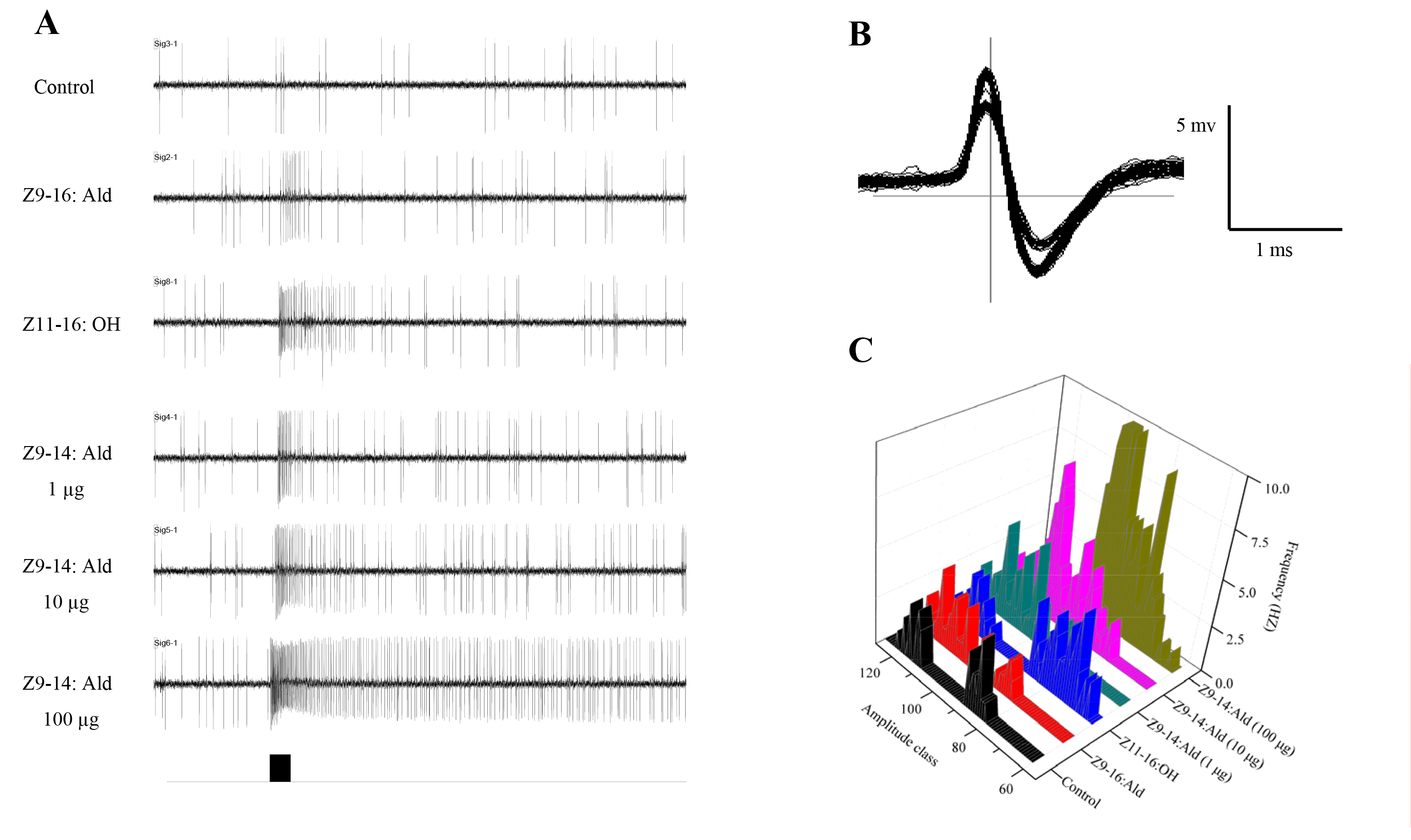

Supplement: Figure S1 — Two types of neurons colocalized within type C sensilla in Helicoverpa armigera. (A) Response profile of type C sensilla to Z9-16:Ald (100 μg), Z11-16:OH (100 μg), and Z9-14:Ald (1, 10, 100 μg). Paraffin oil was used as control. The horizontal black bar indicates stimulus duration of 200 ms. (B) The wave forms of spikes sampled during 10 s of spontaneous activity (same neuron as in A responding to control) indicates two neuron types. (C) Amplitude classification of firing patterns during 10 s showing that Z9-16:Ald activated the larger-spike neuron, Z11-16:OH activated the smaller-spike neuron, while Z9-14:Ald at the dosage 100 μg activated both neurons. [file Image1.TIF]

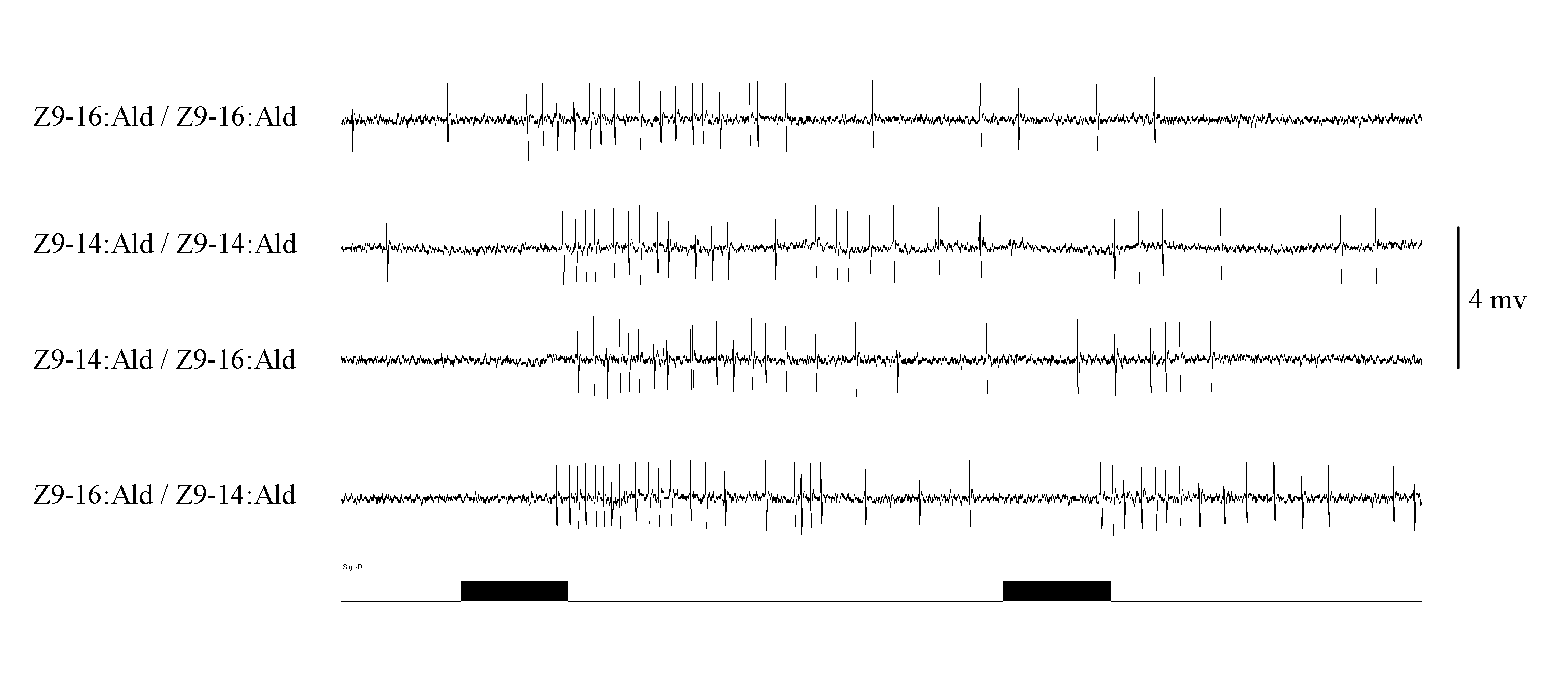

Supplement: Figure S2 — Examples of traces generated during cross-adaptation stimulation of an OSN in a H. armigera Type C sensillum. Horizontal bar denotes stimulus delivery time of 100 ms. The interval between two stimulations is about 350 ms. All stimuli loadings were of 100 μg. [file Image2.TIF]
